# Epidemiology of general obesity, abdominal obesity and related risk factors in urban adults from 33 communities of northeast china: the CHPSNE study

**DOI:** 10.1186/1471-2458-12-967

**Published:** 2012-11-12

**Authors:** Hao Wang, Jing Wang, Miao-Miao Liu, Da Wang, Yu-Qin Liu, Yang Zhao, Mei-Meng Huang, Yang Liu, Jing Sun, Guang-Hui Dong

**Affiliations:** 1 Department of Anesthesiology, The First Affiliated Hospital of Liaoning Medical University, Jinzhou, 121001, P.R. China; 2 Department of Biostatistics, School of Public Health, Saint Louis University, Saint Louis, MO, 63104, USA; 3 Department of Biostatistics and Epidemiology, School of Public Health, China Medical University, Shenyang, 110001, P.R. China

**Keywords:** General obesity, Abdominal obesity, Risk factors, Gender difference, Chinese urban adults

## Abstract

**Background:**

Obesity increases the risk of many diseases. However, there has been little literature about the epidemiology of obesity classified by body mass index (BMI) or waist (abdominal obesity) among urban Chinese adults. This study is to fill the gap by assessing the prevalence of obesity and associated risk factors among urban Chinese adults.

**Methods:**

A representative sample of 25,196 urban adults aged 18 to 74 years in Northeast China was selected and measurements of height, weight and waist circumference (WC) were taken from 2009–2010. Definitions of overweight and obesity by the World Health Organization (WHO) were used.

**Results:**

The overall prevalence rates of general obesity and overweight classified by BMI were 15.0% (15.7% for men and 14.3% for women, *p*<0.01) and 19.2% (20.8% for men and 17.7% for women, *p*<0.01), respectively, and the overall prevalence rate of abdominal obesity was 37.6% (31.1% for men and women 43.9% for women, *p*<0.01). Multivariable logistic regression showed that the elderly and those who had a history of parental obesity, alcohol drinking, or former cigarette smoking were at high risk of obesity classified by BMI or WC, whereas those with a higher level of education, higher family income, or a healthy and balanced diet were at low risk of obesity. Analysis stratified by gender showed that men with a higher level education level, a white-collar job, a cadre job, or higher family income were the high risk group, and women with a higher level of education or higher family income were the low risk group.

**Conclusions:**

Obesity and overweight have become epidemic in urban populations in China; associations of risk factors with obesity differ between men and women.

## Background

Obesity has become a growing global health problem. There are approximately 937 million and 396 million obese and overweight adults worldwide, respectively [1]. China’s rapid economic growth has led to changes in dietary and physical activity patterns, which in turn have led to an increase in obesity prevalence [2-9]. Based on the analysis of the China Health and Nutrition Survey (CHNS) data, Xi et al. reported an increase of 1.2 kg/m^2^ in body mass index (BMI) in those aged above 18 years, an increase of 67% in prevalence of overweight from 9.4% in 1993 to 15.7% in 2009 and an increase of 168% in prevalence obesity from 4.0% in 1993 to 10.7% in 2009, respectively [5]. There have been studies in obesity prevalence in China in general; however, little attention has been given to obesity prevalence in Northeast China. We conjecture that the major proportion of the considerable increase in obesity among Northeast Chinese adults in recent decades may be due to factors such as climate, geography, history, cooking techniques and lifestyle that are distinctive from other regions of China. For example, the interior northeast has very cold weather, especially later on in the winter season compared with other locations, through all months of the year and summer is a brief period of milder climates. Cold weather and shorter days make it harder to exercise outdoors, and therefore there may be more time and temptation for comfort eating and over-indulging at the festivities that surround the Chinese New Year during January-February.

Presently, body mass index (BMI) and waist circumference (WC) are two criteria that have been employed for classifying obesity. Associations of obesity classified by BMI, defined as general obesity, with chronic diseases and reduced life expectancy have been well documented [10]. Based on the data analysis of the 2002 CNHS, Chen et al. reported a 2-5-fold increase in relative risk of adult hypertension and diabetes and a 1.3-2.0-fold increase in relative risk of coronary heart disease and ischaemic stroke in the overweight or obese population measured by body mass index [3]. However, there was evidence of a slowdown of the prevalence of general obesity in some countries [11-14]. In addition, abdominal fat deposition measured by waist circumference (WC) has been suggested as a better indicator of obesity in relation to metabolic syndrome, type 2 diabetes, and cardiovascular diseases than BMI [15-17]. WC has been reported to increase at a faster rate than BMI, and the adverse health consequences of obesity may be underestimated by trends in BMI [18]. Nevertheless, there has been little literature about the prevalence obesity and the related risk factors in Northeast China. To fill this gap, the present study assessed the association of risk factors with the prevalence rates of general obesity and abdominal obesity in the urban Chinese population from 2009–2010, using a sample of 25,196 subjects aged 18–74 years selected from 33 communities in three cities of Northeast China where the prevalence of coronary artery diseases, hypertension, and diabetes was relatively high.

## Methods

### Sample design

**Figure 1:**
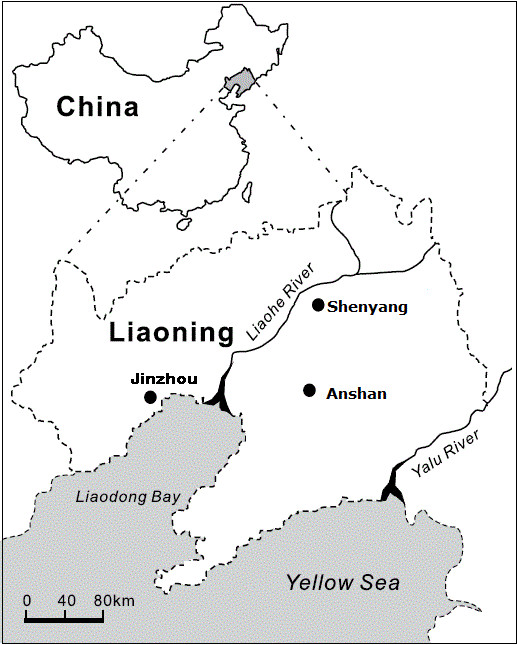
Locations of the study cities in Liaoning province, northeast of China.

Participants in this study were a random sample that was collected from the CHPSNE Study in April 2009 (Control Hypertension and Other Risk Factors to Prevent Stroke with Nutrition Education in Urban Area of Northeast China). The CHPSNE Study used a random representative sample of the general urban population aged 18–74 years. More than 20 million people resided in 14 cities in the Liaoning province in northeast China. These 14 cities were divided into three socioeconomic groups: low, moderate, and high. In April 2009, three cities -Shenyang, Anshan, and Jinzhou- were randomly selected from the three socioeconomic zones. Figure 1 shows the map of these selected cities. The number of urban districts in Shenyang, Anshan and Jinzhou were 5, 3, and 3, respectively. From each of the selected 3 cities, 3 communities were randomly selected from each urban district, yielding a total of 33 communities included in the study. About 1000 households were then randomly selected from each sample community, and from each household one individual with at least 5 years of residency was randomly selected without replacement and then invited to participate in the survey, yielding a total of 28,830 participants. Each participant received a consent form to fill out. A total of 25,196 participants (12,413 men and 12,783 women) aged 18–74 with a mean± standard value of 41.7±14.4 years completed the questionnaire and had a clinical examination done, yielding an overall response rate of 87.4% (86.1% in men and 88.7% in women). Participants who had item non-responses were excluded from the study. This study was approved by the ethics committee of China Medical University.

### Anthropometric measurements and data collection

Anthropometric measurements of height, weight and waist circumference (WC) were obtained using a standardized protocol from each participant during interview and clinical examination [19]. Height was measured, to the nearest 0.5 cm, without shoes, with the participant’s back square against the wall tape, eyes looking straight ahead with a right-angle triangle resting on the scalp and against the wall. Weight was measured with a lever balance to the nearest 100 grams, without shoes, in light undergarments. WC was defined as the midpoint between the lower rib and upper margin of the iliac crest, measured by a nurse using a tape with an insertion buckle at one end. WC was measured to the nearest 0.5 cm. Body mass index (BMI) was calculated by dividing weight (in kilograms) by height (in meters) squared (kg/m^2^). Using the BMI classifications for Chinese by the WHO, a BMI ≥ 25 kg/m^2^ and < 27.5 kg/m^2^ was classified as overweight and a BMI ≥ 27.5 kg/m^2^ as general obese [20]. Using the WC criterion, a WC ≥ 90 cm in men or WC ≥ 80 cm in women was classified as abdominal obese [21]. In addition, to ensure comparability with other studies, our study also incorporated the criteria recommended by the Working Group on Obesity in China (WGOC) (overweight: 24 kg/m^2^ ≤ BMI < 28 kg/m^2^; general obese: BMI ≥ 28 kg/m^2^; abdominal obesity: WC ≥ 85 cm for men and ≥ 80 cm for women) [22] and the WHO classifications for Europids (overweight: 25 kg/m^2^ ≤ BMI < 30 kg/m^2^; general obese: BMI ≥ 30 kg/m^2^; abdominal obese: WC ≥ 102 cm for men and ≥ 88 cm for women) [23] . Information on demographics (i.e., age, gender and residential area), socio-economic status (i.e., education level achieved, occupation and annual household income), and other information was collected through a questionnaire.

### Statistical methods

The mean ± SD value were calculated for continuous variables and the percentage of each subgroup for categorical variables. Comparisons between two group means were performed by using the *t-* test. Associations between categorical variables were tested by using contingency tables and the χ^2^ test. Adjusted odds ratios (ORs) with the associated 95% confidence intervals (95% CIs) for general obesity and abdominal obesity were calculated from multivariate logistic regression. All data analyses were conducted using SAS statistical software (Version 9.1; SAS Institute Inc., Cary, NC, USA). All statistical tests were 2-tailed, and *p* values <0.05 were considered statistically significant.

## Results

### Demographic characteristics

Distributions of the characteristics of 12413 men and 12783 women are shown in Table 1. Participants aged from 18–74 years and their average ages were 41.2 ± 14.5 for men and 42.2 ± 14.4 years for women, respectively. Men have higher mean BMI and WC values (24.2 ± 3.8 kg/m^2^ for BMI and 84.0 ± 10.9 cm for WC) than women (23.5 ± 3.7 kg/m^2^ for BMI and 78.8 ±10.6 cm for WC).

**Table 1:** Distributions of demographics and lifestyle factors by gender

**Variables**	**Total n (%)**	**Men n (%)**	**Women n (%)**	***p *values**^**c**^
Age (years) ^a^	41.7±14.4	41.2±14.5	42.2±14.4	<0.001
BMI (kg/m^2^) ^a^	23.8±3.7	24.2±3.8	23.5±3.7	<0.001
WC (cm) ^a^	81.4±11.1	84.0±10.9	78.8±10.6	<0.001
Ethnic				
Han	23169 (92.0)	11179 (90.1)	11990 (93.8)	<0.001
Others	2027 (8.0)	1234 (9.9)	793 (6.2)	
Educational level				
No school	983 (3.9)	177 (1.4)	806 (6.3)	<0.001
Primary school	3225 (12.8)	1314 (10.6)	1911 (15.0)	
Junior high school	14736 (58.5)	7434 (59.9)	7302 (57.1)	
≥Senior high school	6252 (24.8)	3488 (28.1)	2764 (21.6)	
Occupation				
Blue-collar worker	11887 (47.2)	4732 (38.1)	7155 (56.0)	<0.001
White-collar worker	4011 (15.9)	2198 (17.7)	1813 (14.2)	
Cadres	1894 (7.5)	1188 (9.6)	706 (5.5)	
Others	7404 (29.4)	4295 (34.6)	3109 (24.3)	
Family income/year ^b^				
<10,000 RMB	2484 (9.9)	1376 (11.1)	1108 (8.7)	<0.001
10,000-30,000 RMB	3286 (13.0)	1275 (10.3)	2011 (15.7)	
30,000-50,000 RMB	13004 (51.6)	6522 (52.5)	6482 (50.7)	
>50,000RMB	6422 (25.5)	3240 (26.1)	3182 (24.9)	
Parental obesity				
No	20178 (80.1)	9775 (78.8)	10363 (81.1)	<0.001
Yes	5018 (19.9)	2638 (21.2)	2420 (18.9)	
Physical activity				
Low	18207 (72.3)	8638 (69.6)	9569 (74.9)	<0.001
High	6989 (27.7)	3775 (30.4)	3214(25.1)	
Cigarette smoking				
Never	17180 (68.2)	5256 (42.3)	11924 (93.2)	<0.001
Current	7094 (28.1)	6356 (51.2)	738 (5.8)	
Former	922 (3.7)	801 (6.5)	121 (1.0)	
Alcohol consumption				
Never	19240 (76.4)	6849 (55.2)	12391 (96.9)	<0.001
1 drink per day	842 (3.3)	788 (6.3)	54 (0.4)	
≥2 drinks per day	5114 (20.3)	4776 (38.5)	338 (2.7)	
Eat fried foods				
Never	19820 (78.7)	8675 (69.9)	11145 (87.2)	<0.001
Yes	5376 (21.3)	3738 (30.1)	1638 (12.8)	
Have a diet low in fat/calorie				
No	17365 (68.9)	8015 (64.6)	9350 (73.1)	<0.001
Yes	7831 (31.1)	4398 (35.4)	3433 (26.9)	

^a^ Data are mean ± SD; BMI, Body mass index; WC, Waist circumference; RMB, Chinese yuan.

^b^ 100 RMB=6.35 US $.

^c^*p* values are calculated for gender difference by *t*-test or χ^2^ test.

Table 2 shows the prevalence of overweight, general obesity and abdominal obesity by the WHO classifications for Chinese and Europids. Using the WHO classifications for Chinese, the overall prevalence of general obesity and overweight were 15.0% (15.7% for men and 14.3% for women, *p*<0.01) and 19.2% (20.8% for men and 17.7% for women, *p*<0.01), respectively, and the overall prevalence of abdominal obesity was 37.6% (31.1% for women and 43.9% for women, *p*<0.01). The age distributions for men and women differed within each BMI or WC category. The prevalence of overweight, general obesity and abdominal obesity by the WGOC criteria for Chinese are depicted in Additional file 1: Table S1.

**Table 2:** Prevalence rates of Overweight and Obesity Classified by BMI and WC by Age and Gender

**Age (years)**	**n**	**BMI (kg/m**^**2**^**)**				**WC (cm)**	
		**Overweight**^**a**^	**Obesity**^**a**^	**Overweight**^**b**^	**Obesity**^**b**^	**Abdominal obesity**^**a**^	**Abdominal obesity**^**b**^
Men							
18-34	5028	1044 (20.8)	888 (17.7)	1584 (31.5)	348 (6.9)	1452 (28.9)	180 (3.6)
35-44	2560	507 (19.8)	405 (15.8)	778 (30.4)	134 (5.2)	809 (31.6)	162 (6.3)
45-54	2469	570 (23.1)	360 (14.6)	837 (33.9)	93 (3.8)	856 (34.7)	117 (4.7)
55-64	1426	298 (20.9)	211 (14.8)	461 (32.3)	48 (3.4)	479 (33.6)	75 (5.3)
65-74	930	163 (17.5)	90 (9.7)	224 (24.1)	29 (3.1)	268 (28.8)	46 (5.0)
Total	12413	2582 (20.8)	1954 (15.7)	3884 (31.3)	652 (5.3)	3864 (31.1)	580 (4.7)
Women							
18-34	4792	496 (10.4)	360 (7.5)	704 (14.7)	152 (3.2)	1096 (22.9)	400 (8.4)
35-44	2693	473 (17.6)	326 (12.1)	707 (26.3)	92 (3.4)	1108 (41.1)	395 (14.7)
45-54	2689	708 (26.3)	526 (19.6)	1066 (39.6)	168 (6.3)	1556 (57.9)	644 (24.0)
55-64	1670	375 (22.5)	468 (28.0)	649 (38.9)	194 (11.6)	1225 (73.4)	746 (44.7)
65-74	939	211 (22.5)	150 (16.0)	308 (32.8)	53 (5.6)	627 (66.8)	320 (34.1)
Total	12783	2263 (17.7)	1830 (14.3)	3434 (26.9)	659 (5.2)	5612 (43.9)	2505 (19.6)
All subjects							
18-34	9820	1540 (15.7)	1248 (12.7)	2288 (23.3)	500 (5.1)	2548 (26.0)	580 (5.9)
35-44	5253	980 (18.7)	731 (13.9)	1485 (28.3)	226 (4.3)	1917 (36.5)	557 (10.6)
45-54	5158	1278 (24.8)	886 (17.2)	1903 (36.9)	261 (5.1)	2412 (46.8)	761 (14.8)
55-64	3096	673 (21.7)	679 (21.9)	1110 (35.9)	242 (7.8)	1704 (55.0)	821 (26.5)
65-74	1869	374 (20.0)	240 (12.8)	532 (28.5)	82 (4.4)	895 (47.9)	366 (19.6)
Total	25196	4845 (19.2)	3784 (15.0)	7318 (29.0)	1311 (5.2)	9476 (37.6)	3085 (12.2)

Data are n (%). BMI, Body mass index; WC, Waist circumference.

^a^ Using WHO criteria for Chinese (Overweight: 25 kg/m^2^≤BMI<27.5 kg/m^2^; Obesity: BMI≥27.5 kg/m^2^; abdominal obesity: WC≥90 cm for men, WC≥80 cm for women).

^b^ Using WHO criteria for Europids (Overweight: 25 kg/m^2^≤BMI<30 kg/m^2^; Obesity: BMI≥30 kg/m^2^; abdominal obesity: WC≥102 cm for men, WC≥88 cm for women).

The results from multiple logistic regression analysis on general obesity and abdominal obesity by the WHO criteria for Chinese in men and women, respectively, are shown in Additional file 2: Table S2. Men had a higher risk of general obesity (OR=1.42, 95% CI: 1.29-1.55), and a lower risk of abdominal obesity than women (OR=0.65, 95% CI: 0.61-0.70). History of parents obesity was associated with an increased risk of both general obesity (OR=2.61, 95% CI: 2.40-2.83) and abdominal obesity (OR=1.82, 95% CI 1.70-1.95). White-collar workers and cadres had a higher risk of general obesity or abdominal obesity than blue-collar workers. A higher level of education, higher income, consuming no more than 1 drink per day, and having a diet low in fat/calories were each associated with a lower risk of general obesity and abdominal obesity.

Results stratified by gender showed differences in association of risk factors with obesity between men (Table 3) and women (Table 4). Among men, the association between age and the prevalence of general obesity was not significant, and a higher level of education and higher income had a higher risk of obesity (Table 3). By contrast, among women an older age was associated with an increased risk of general obesity prevalence, and a higher level of education and higher income were each associated with a lower obesity prevalence (Table 4). In addition, there was an association between occupation and obesity prevalence in men (Table 3 and Table 4).

**Table 3:** Factors Associated with the Crude Prevalence of Obesity from Multivariate Logistic Regression Models in Men (N=12,413)

**Variables**	**General obesity**^**b**^		**Abdominal obesity**^**b**^	
	**(%)**	**OR (95% CI)**^**a**^	**(%)**	**OR (95% CI)**^**a**^
Age-groups (years)				
18-34	17.7	1.00	28.9	1.00
35-44	15.8	1.19(1.03-1.37)	31.6	1.50(1.33-1.68)
45-54	14.6	1.09(0.94-1.27)	34.7	1.77(1.57-2.00)
55-64	14.8	1.29 (1.06-1.56)	33.6	1.87(1.61-2.17)
65-74	9.7	0.88(0.67-1.14)	28.8	1.60(1.33-1.92)
Ethnic				
Han	16.1	1.00	31.6	1.00
Others	12.7	0.83(0.69-0.99)	26.6	0.86(0.75-0.99)
Educational level				
No school	9.0	1.00	20.3	1.00
Primary school	9.7	1.42(0.80-2.52)	22.0	1.50(1.00-2.25)
Junior high school	14.6	1.92(1.11-3.31)	31.6	2.36(1.60-3.48)
≥Senior high school	20.8	2.45(1.40-4.29)	34.1	2.39(1.60-3.56)
Occupation				
Blue-collar	13.9	1.00	27.9	1.00
White-collar	20.9	1.31(1.12-1.52)	40.0	1.64(1.45-1.85)
Cadres	16.7	1.33(1.11-1.61)	34.9	1.57(1.36-1.82)
Others	14.9	1.34(1.18-1.52)	29.2	1.19(1.08-1.31)
Family income/year ^c^				
<10,000 RMB	12.5	1.00	27.9	1.00
10,000-30,000 RMB	13.8	0.91(0.72-1.15)	32.1	1.07(0.90-1.27)
30,000-50,000RMB	14.1	0.78(0.65-0.94)	30.0	0.94(0.82-1.08)
>50,000RMB	21.1	1.05(0.86-1.29)	34.4	1.04(0.89-1.21)
Parental obesity				
No	12.2	1.00	27.5	1.00
Yes	34.4	3.51(3.13-3.94)	50.4	2.72 (2.45-3.02)
Physical activity				
Low	15.1	1.00	30.4	1.00
High	18.0	0.98(0.87-1.10)	33.7	1.00(0.90-1.10)
Cigarette smoking				
Never	16.8	1.00	31.7	1.00
Current	13.6	0.81(0.72-0.91)	28.9	0.85(0.78-0.93)
Former	25.2	1.81(1.50-2.18)	45.1	1.61(1.37-1.88)
Alcohol consumption				
Never	15.4	1.00	29.7	1.00
1 drink per day	10.7	0.84(0.65-1.09)	27.7	0.91(0.76-1.09)
≥2 drinks per day	17.1	1.16(1.04-1.30)	33.7	1.22(1.11-1.33)
Eat fried foods				
Never	17.5	1.00	33.7	1.00
Yes	11.6	0.79(0.70-0.89)	25.3	0.85(0.78-0.93)
Have a diet low in fat/calories				
No	17.6	1.00	34.0	1.00
Yes	12.4	0.75(0.67-0.84)	25.9	0.64(0.58-0.70)

^a^ Logistic regression models were used to adjust for all other variables in the table. CI, confidence interval; OR, odds ratio.

^b^ Using WHO criteria for Chinese (general obesity: BMI≥27.5 kg/m^2^; abdominal obesity: WC≥90 cm for men, WC≥80 cm for women).

^c^ 100 RMB (Chinese yuan)=6.35 US $.

**Table 4:** Risk Factors Associated with the Crude Prevalence of Obesity from Multivariate Logistic Regression in Women (N=12,783)

**Variables**	**General obesity**^**b**^		**Abdominal obesity**^**b**^	
	**(%)**	**OR (95% CI)**^**a**^	**(%)**	**OR (95% CI)**^**a**^
Age-groups (years)				
18-34	7.5	1.00	22.9	1.00
35-44	12.1	1.45(1.23-1.71)	41.1	1.96(1.76-2.18)
45-54	19.6	2.57(2.19-3.01)	57.9	3.59(3.21-4.02)
55-64	28.0	4.39(3.68-5.24)	73.4	6.85(5.93-7.91)
65-74	16.0	2.57(2.01-3.29)	66.8	4.96 (4.11-5.98)
Ethnic				
Han	14.6	1.00	45.0	1.00
Others	10.1	0.86(0.67-1.10)	27.7	0.55(0.46-0.66)
Educational level				
No school	19.0	1.00	64.8	1.00
Primary school	20.9	1.13(0.91-1.41)	65.5	1.33(1.10-1.60)
Junior high school	15.0	1.02(0.82-1.27)	43.6	0.96(0.80-1.15)
≥Senior high school	6.6	0.61(0.45-0.81)	23.6	0.61(0.49-0.75)
Occupation				
Blue-collar	15.3	1.00	46.6	1.00
White-collar	9.4	0.77(0.63-0.94)	36.9	1.12(0.98-1.28)
Organization	12.6	1.19(0.93-1.54)	37.4	1.17(0.97-1.42)
Others	15.3	0.93(0.82-1.06)	43.3	0.81(0.74-0.90)
Family income/year ^c^				
<10,000 RMB	18.7	1.00	65.1	1.00
10,000-30,000 RMB	17.5	1.09(0.90-1.34)	55.1	0.83(0.70-0.98)
30,000-50,000RMB	13.9	1.00(0.83-1.20)	42.8	0.62(0.53-0.72)
>50,000RMB	11.6	1.02(0.82-1.26)	31.7	0.47(0.40-0.56)
Parental obesity				
No	13.0	1.00	43.8	1.00
Yes	20.3	2.06(1.82-2.33)	44.5	1.26(1.14-1.40)
Physical activity				
Low	14.1	1.00	42.1	1.00
High	15.0	0.89 (0.79-1.01)	49.3	0.99(0.90-1.09)
Cigarette smoking				
Never	14.5	1.00	43.2	1.00
Current	11.0	0.47(0.36-0.60)	50.0	0.58(0.49-0.68)
Former	13.2	0.63(0.36-1.08)	71.9	2.31(1.47-3.62)
Alcohol consumption				
Never	14.4	1.00	43.7	1.00
1 drink per day	7.4	0.32(0.11-0.90)	63.0	0.79(0.44-1.41)
≥2 drinks per day	11.0	0.79(0.55-1.13)	46.8	1.46(1.15-1.86)
Eat fried foods				
Never	15.3	1.00	46.1	1.00
Yes	7.6	0.63(0.51-0.77)	29.2	0.76(0.67-0.86)
Have a diet low in fat/calories				
No	15.2	1.00	43.1	1.00
Yes	11.9	0.67(0.59-0.76)	46.1	1.05(0.96-1.15)

^a^ Logistic regression models were used to adjust for all other variables in the table. CI, confidence interval; OR, odds ratio.

^b^ Using WHO criteria for Chinese (general obesity: BMI≥27.5 kg/m^2^; abdominal obesity: WC≥90 cm for men, WC≥80 cm for women).

^c^ 100 RMB (Chinese yuan)=6.35 US $.

## Discussion

This study was conducted on a large representative sample of urban adults in Northeast China using standard protocols and instruments. Data collectors were trained rigorously to ensure the quality of data collection. Another major strength of the study was the high response rate. The large sample size helped to ensure statistical accuracy and reliability of the assessment of associations of risk factors with the prevalence rates of overweight and obesity classified by BMI and WC.

### General obesity

It is difficult to compare the obesity prevalence between this study and previous studies because of the difference in age distributions defined in these studies. The present study indicated an overall prevalence rate of 15.0% for general obesity and 19.2% for overweight and 34.2% for both general obesity and overweight in the population aged 18–74 years in Northeast of China, which were relatively high to other regions of China. The prevalence of obesity, defined as BMI≥30 kg/m^2^, from a large cross-sectional population survey on 58,308 residents aged over 40 years in Beijing was reported as 15.1% (10.7% for men and 17.5% for women) [24]. The prevalence rates of obesity, defined as BMI≥30 kg/m^2^, from the study on adult age 40 years or above in Shanghai were reported as 7.4% for women and 4.8% for men [25]. Using a cut point of BMI≥27.5 kg/m^2^, the prevalence of obesity among Hong Kong Chinese population aged 18–81 years was 14.0% in men and 9.5% in women [26]. From another study we conducted recently on rural populations of Northeast China, the combined prevalence of overweight or general obesity in adults aged 35–99 years was found to be 20.3% [27]. In the 1993–2009 CHNS study on 52621 Chinese adults aged above 18 years, the prevalence rates of overweight, defined as 25≤BMI<27.5 kg/m^2^, and general obesity, defined as BMI≥27.5 kg/m^2^,were found to be 17.1% and 11.4% in men, and 14.4% and 10.1% in women in 2009, respectively [5]. The prevalence rates of obesity, defined as BMI ≥ 30 kg/m^2^ from the 2005–2008 NAHSIT III study on Taiwanese adults aged 18 years or above were reported as 6.1% and 6.4% in men and women, respectively [28]. Over the past decade, China has seen rapid economic growth that had led to changes in dietary physical activity patterns and an increase in life expectancy, which in turn has led to an increase in obesity prevalence at an alarming rate in China. Based on the China Nationwide Nutrition and Health Survey data, the combined prevalence of overweight or obesity (BMI≥25 kg/m^2^) has increased by 97%, from 13.4% in 1993 to 26.4% in 2009 [5]. Compared with the results of the China Nationwide Nutrition and Health Survey, the present study reinforced that obesity has become a major public health problem in the urban population in Northeast China, and therefore there is an urgent need for obesity prevention strategies.

### Abdominal obesity

This study reported the prevalence of abdominal obesity as classified by the WHO criteria for Chinese (WC ≥ 90 cm for men and WC ≥ 80 cm for women) was 37.6% (men: 31.1% and women: 43.9%) in the urban population aged 18–74 in Northeast China. Based on the CHNS study data, Xi et al. reported abdominal obesity prevalence rates of 27.8% among men and 45.9% among women [5]. The prevalence of abdominal obesity, defined as WC ≥ 90 cm for men and WC ≥ 80 cm for women, from the Shanghai’s study described earlier were reported as 25.5% for men and 42.6% for women [25]. The prevalence rates of abdominal obesity (defined as WC ≥ 90 cm for men, and WC ≥ 80 cm for women) from a study on adults aged 18–81 years in Hong Kong were reported as 28.0% for men and 28.3% for women, respectively [26]. The prevalence rates of abdominal obesity from the 2000–2001 International Collaborative Study of Cardiovascular disease in Asians aged 35–74 years were reported as 16.1% (27.3% for urban areas and 13.4% for rural areas ) for men (WC ≥90 cm) and 37.6% (43.8% for urban areas and 36.6% for rural areas ) for women (WC ≥ 80 cm) [29]. The Heart Outcomes Prevention Evaluation (HOPE) study reported that abdominal adiposity reduced the prognosis of patients with cardiovascular disease (CVD) [30]. It has been suggested that abdominal fat depositions measured by WC is a better indicator of the risks of metabolic syndrome, type 2 diabetes and cardiovascular disease than general obesity [15-17].

### Risk factors

Age is strongly associated with obesity prevalence. In many studies, the prevalence of obesity has been reported to increase with age [31-33]. In our study, except for general obesity in men, the associations between age and general obesity, abdominal obesity prevalence firstly increased and then decreased with age. The prevalence of obesity was highest in women aged 55–64 years (28.0% for general obesity, 73.4% for abdominal obesity) and in men aged 45–54 years (34.7% for abdominal obesity). Such pattern observed from obesity and age may be explained, in part, by the decrease in physical activity level with age [33]. Furthermore, women are also prone to weight gain during menopause because the loss of menstrual cycle may affect calorie intake and slightly lowers metabolic consumption [34]. The subsequent decline in obesity with age has also been documented in other populations [35,36] and may be partly due to the survivor effect as those people more succumb to obesity-related diseases, die at an earlier age than those who have been able to control their weight [37]. Interestingly, contrast to the patterns in women, there was a consistent decreases in general obesity prevalence with the age in men. However, when adjusted for other covariables, the OR for general obesity striking increased to 1.29 (95% CI: 1.06-1.56) for men aged 55–64 years, whereas the prevalence of obesity was only 14.8% in this age group as compared to the obesity prevalence of 17.7% in men ages 18–34 years. The reasons for the general obesity and age association in men are still quite unclear, future studies are therefore warranted.

The disproportionately elevated and steadily increasing rates of overweight and obesity among different ethnic minority communities have also been reported. For example, the prevalence of obesity varies markedly by ethnicity, ranging from 27% (Mexican American men) to 49% (black women) in the United States [38]. A study in China showed a difference in prevalence of obesity among Han, Hui, Uygur, and Kazak Chinese; with the obesity prevalence being highest in Han boys and Hui girls and lowest in Kazak boys and girls [39]. In our study, Han Chinese appeared to have a higher risk of being obesity than other Chinese ethnic groups. Such differences in obesity prevalence among Chinese ethnic groups may be attributable to the difference in genetic and lifestyle.

The founding from the present study also indicated that subjects of obese parents had a substantially higher risk of obesity than subjects with no obese parents. There has been intensive literature about such positive association between obesity and genetic factors [40], prenatal factors [41] or postnatal environmental factors [42]. Consistent with many prior studies [43], general obesity and abdominal obesity were higher among low educated individuals, and higher education status was shown to be a protective factor after adjustment for other confounders. Compared to people with no schooling, people with some schooling may have had more opportunities to gain information about obesity and develop healthy lifestyle habits, so that their prevalence was low. A similar pattern was also observed with family income. Whereas, working in organization cadres appears to be associated with a higher risk of developing obesity, especially for abdominal obesity, than blue-collar work in men, which may suggest an association between obesity and the risk factors differences between organization cadres and blue-collar workers. For example, people who work in organization cadres tend to spend more time in front of their computers and less time exercising and/or may suffer from mental health problems due to work-related stress. In addition, in the present study, 57.9% of organization cadre personnel were men, and the average age of organization cadre personnel (40.5±15.4 years) was higher than that of blue-collar workers (37.8±13.3 years).

The association between smoking and overweight was found negative from this study. We have noticed a large percentage of male smokers in this study; therefore a possible explanation of such a negative association might be partly due to the negative effects of smoking on metabolic rate, energy intake and storage, and energy expenditure [44,45]. However, another study has reported that weight gain was observed in current smokers, ex-smokers, and non-smokers, which seemed to indicate that factors that promote weight gain had overcome the inverse effect of smoking [46]. Although this study found non-smokers had the greatest risk of obesity, smoking has been previously reported to have a greater impact on morbidity and mortality than obesity [47,48].

There has been evidence that the global epidemic of obesity was mainly related to societal factors that promote a sedentary lifestyle and consumption of high-fat, energy-dense foods [21]. A previous study in China showed that a high intake of energy and carbohydrate, a high intake of protein and fat by men, a low rate of physically-active commuting and low occupational activity were associated with a higher incidence of overweight [49]. Our study also found that high-fat/calories foods and drinking increased the risk of obesity, whereas food low in fat/calories decreased the obesity risk. Similar findings were also obtained from the ATTICA Study [50] and the studies from Australia and America [51,52].

### Gender difference

Many developed countries have seen a high prevalence of obesity in both men and women; however, it has been reported that in many countries with a relatively low gross national product women had a obesity prevalence approximately 1.5-2 times higher than men [53]. We found from our study that men had a higher prevalence of overweight or general obesity than women, whereas women had a higher prevalence of abdominal obesity than men. Similar findings were also obtained from a study in Japan where obesity was more prevalent in men than in women [54]. A study in Iran reported that the prevalence of abdominal obesity was found to be higher in women (53.5%) than in men (12.5%) [43]. We hypothesize the variability in the difference in prevalence of obesity between men and women around the world might be partly explained by the difference in lifestyle, socio-demographic, genetic or behavioral characteristics.

Gender is understood to be a social construct, and the gender role is a set of social and behavioral norms in women and men [55]. In our study, multivariable logistic regression analysis revealed that the associations of risk factors with the prevalence of obesity were different among men and women. As shown in Table 3 and Table 4, associations of the prevalence of obesity and the level of education, income were both positive among men but negative among women. White-collar male workers had a higher prevalence of general obesity (OR=1.31, 95% CI: 1.12-1.52) than blue-collar male workers, whereas female white-collar workers were found to have a lower risk of general obesity (OR=0.77, 95% CI: 0.63-0.94) than female white-collar workers. Education level is a major difference in blue-collar and white-collar jobs, and white-collar work generally requires formal education; therefore a possible explanation of the high obesity prevalence in male while-collar workers might be related to the potential risk factors such as work-related stress and anxiety. Further studies are warranted to gain more insight into such association.

Our study has several limitations. The most obvious limitation is its cross-sectional design, which can not infer causal relationships between obesity and socio-demographic lifestyle characteristics. Second, because exposure information was primarily obtained through a questionnaire, recall bias, where the respondent might be affected not just by the correct answer but also by the respondent’s memory, can not be ruled out. Third, due to the nature of the survey questions (yes or no), misclassification of behavioral risk factors (occupation, family income, smoking, drinking, and exercising) were possible. Finally, as noted by previous studies, a significant association has been found between obesity and confounding factors such as marriage age, reproductive history (higher parity and earlier age at menarche), menopause, family structure, medication use, chronic disease status and obesity [37,56], however, these confounding factors are not available in the present study.

## Conclusions

In conclusion, the prevalence of overweight and obesity have become unacceptably high in urban Chinese adults. Abdominal obesity was more prevalent in women than in men. Prevention strategies that take account of differences in association of risk factors with obesity between women and men from culture to culture are therefore needed with great urgency.

## Abbreviations

OR: Odds ratio; 95% CI: 95% confidence interval; BMI: Body mass index; WC: Waist circumference; CVD: Cardiovascular disease.

## Competing interests

Authors report no conflicts of interest. Authors are alone responsible for the content and writing of the paper.

## Authors’ contributions

Conceived and designed the experiments: HW GHD. Performed the experiments: HW. Analyzed the data: GHD, JW. Contributed reagents/materials/analysis tools: HW MML DW YQL YZ MMH YL JS. Wrote the paper: HW. Revised the paper: GHD, JW. Contributed the investigation: MML DW YQL YZ MMH YL JS. All authors read and approved the final manuscript.

## Pre-publication history

The pre-publication history for this paper can be accessed here:

http://www.biomedcentral.com/1471-2458/12/967/prepub

## Supplementary Material

Additional file 1 **Table S1.** Prevalence of Overweight and Obesity Based on the Distribution of BMI and WC with Respect to Age Groups and Gender by using the Working Group on Obesity in China (WGOC) criteria. Click here for file

Additional file 2 **Table S2.** Factors Associated with the Crude Prevalence of Obesity from Multivariate Logistic Regression Models in Urban Adults (N=25,196). Click here for file
