# Supplementary material for: Epidemiology of general obesity, abdominal obesity and related risk factors in urban adults from 33 communities of northeast china: the CHPSNE study
Source: BMC Public Health. 2012 Nov 12;12:967. doi: 10.1186/1471-2458-12-967 (PMC3509037; doi:10.1186/1471-2458-12-967)
Supplement: Additional file 1 — Table S1. Prevalence of Overweight and Obesity Based on the Distribution of BMI and WC with Respect to Age Groups and Gender by using the Working Group on Obesity in China (WGOC) criteria. [file 1471-2458-12-967-S1.doc]

| Additional Table 1 The prevalence of overweight, BMI-derived obesity and abdominal obesity among Chinese adults from northeast China (using WGOC criteria) | | | | | |
| --- | --- | --- | --- | --- | --- |
| Age (years) | n | Overweight | BMI-derived Obesity |  | Abdominal obesity |
|  |  | (24 kg/m^2^≤BMI<28 kg/m^2^) | (BMI≥28 kg/m^2^) |  | (WC≥85cm for men,  WC≥80 for women) |
| Men |  |  |  |  |  |
| 18-34 | 5028 | 1620 (32.2) | 780 (15.5) |  | 2100 (41.8) |
| 35-44 | 2560 | 953 (37.2) | 318 (12.4) |  | 1277 (50.0) |
| 45-54 | 2469 | 969 (39.3) | 296 (12.0) |  | 1324 (53.6) |
| 55-64 | 1426 | 492 (34.5) | 183 (12.8) |  | 763 (53.5) |
| 64-74 | 930 | 292 (31.4) | 72 (7.7) |  | 441 (47.4) |
| Total | 12413 | 4326 (34.9) | 1649 (13.3) |  | 5905 (47.6) |
| Women |  |  |  |  |  |
| 18-34 | 4792 | 872 (18.2) | 304 (6.3) |  | 1096 (22.9) |
| 35-44 | 2693 | 824 (30.6) | 246 (9.1) |  | 1108 (41.1) |
| 45-54 | 2689 | 1082 (40.2) | 432 (16.1) |  | 1556 (57.9) |
| 55-64 | 1670 | 667 (39.9) | 379 (22.7) |  | 1225 (73.4) |
| 64-74 | 939 | 341 (36.3) | 125 (13.3) |  | 627 (66.8) |
| Total | 12783 | 3786 (29.6) | 1486 (11.6) |  | 5612 (43.9) |
| All subjects | |  |  |  |  |
| 18-34 | 9820 | 2492 (25.4) | 1084 (11.0) |  | 3196 (32.6) |
| 35-44 | 5253 | 1777 (33.8) | 564 (10.7) |  | 2385 (45.4) |
| 45-54 | 5158 | 2051 (39.8) | 728 (14.1) |  | 2880 (55.8) |
| 55-64 | 3096 | 1159 (37.4) | 562 (18.2) |  | 1988 (64.2) |
| 64-74 | 1869 | 633 (33.9) | 197 (10.5) |  | 1068 (57.1) |
| Total | 25196 | 8112 (32.2) | 3135 (12.4) |  | 11517 (45.7) |

Data are n (%). BMI, Body mass index; WC, Waist circumference
