# Supplementary material for: Epidemiology of general obesity, abdominal obesity and related risk factors in urban adults from 33 communities of northeast china: the CHPSNE study
Source: BMC Public Health. 2012 Nov 12;12:967. doi: 10.1186/1471-2458-12-967 (PMC3509037; doi:10.1186/1471-2458-12-967)
Supplement: Additional file 2 — Table S2. Factors Associated with the Crude Prevalence of Obesity from Multivariate Logistic Regression Models in Urban Adults (N=25,196). [file 1471-2458-12-967-S2.doc]

| Additional Table 2 Factors Associated with the Crude Prevalence of Obesity from Multivariate Logistic Regression Models in Urban Adults (N=25,196) | | | | | |
| --- | --- | --- | --- | --- | --- |
| Variables | General obesity ^b^ | |  | Abdominal obesity ^b^ | |
|  | (%) | OR (95%CI) ^a^ |  | (%) | OR (95%CI) ^a^ |
| Age-groups (years) |  |  |  |  |  |
| 18-34 | 12.7 | 1.00 |  | 26.0 | 1.00 |
| 35-44 | 13.9 | 1.23(1.11-1.37) |  | 36.5 | 1.62(1.50-1.75) |
| 45-54 | 17.2 | 1.58(1.43-1.76) |  | 46.8 | 2.38(2.20-2.58) |
| 55-64 | 21.9 | 2.36(2.09-2.67) |  | 55.0 | 3.41(3.09-3.76) |
| 65-74 | 12.8 | 1.31(1.10-1.55) |  | 47.9 | 2.56(2.26-2.90) |
| Gender |  |  |  |  |  |
| Female | 14.3 | 1.00 |  | 43.9 | 1.00 |
| Male | 15.7 | 1.42(1.29-1.55) |  | 31.1 | 0.65(0.61-0.70) |
| Ethnic |  |  |  |  |  |
| Han | 15.3 | 1.00 |  | 38.5 | 1.00 |
| Others | 11.7 | 0.83(0.72-0.96) |  | 27.0 | 0.71(0.63-0.79) |
| Educational level |  |  |  |  |  |
| No school | 17.2 | 1.00 |  | 56.8 | 1.00 |
| Primary school | 16.3 | 0.95(0.78-1.16) |  | 47.8 | 0.93(0.80-1.08) |
| Junior high school | 14.8 | 0.86(0.71-1.04) |  | 37.6 | 0.79(0.68-0.92) |
| ≥Senior high school | 14.5 | 0.88(0.71-1.09) |  | 29.5 | 0.68(0.57-0.80) |
| Occupation |  |  |  |  |  |
| Blue-collar | 14.8 | 1.00 |  | 39.1 | 1.00 |
| White-collar | 15.7 | 1.01(0.90-1.14) |  | 38.6 | 1.28(1.17-1.40) |
| Cadres | 15.2 | 1.13(0.98-1.32) |  | 35.8 | 1.21(1.08-1.35) |
| Others | 15.1 | 1.07(0.98-1.17) |  | 35.1 | 0.95(0.89-1.02) |
| Family income/year ^c^ |  |  |  |  |  |
| <10,000 RMB | 15.3 | 1.00 |  | 44.5 | 1.00 |
| 10,000-30,000 RMB | 16.0 | 0.99(0.86-1.15) |  | 46.1 | 0.98(0.88-1.10) |
| 30,000-50,000RMB | 14.0 | 0.89(0.78-1.01) |  | 36.4 | 0.79(0.71-0.86) |
| >50,000RMB | 16.4 | 1.06(0.91-1.22) |  | 33.1 | 0.75(0.67-0.84) |
| Parental obesity |  |  |  |  |  |
| No | 12.6 | 1.00 |  | 35.6 | 1.00 |
| Yes | 26.7 | 2.61(2.40-2.83) |  | 47.2 | 1.82(1.70-1.95) |
| Physical activity |  |  |  |  |  |
| Low | 14.6 | 1.00 |  | 36.3 | 1.00 |
| High | 16.4 | 1.02(0.94-1.12) |  | 41.9 | 1.15(1.07-1.22) |
| Cigarette smoking |  |  |  |  |  |
| Never | 15.2 | 1.00 |  | 39.7 | 1.00 |
| Current | 13.4 | 0.73(0.66-0.80) |  | 31.1 | 0.81(0.75-0.87) |
| Former | 23.6 | 1.40(1.18-1.66) |  | 48.6 | 1.50(1.30-1.74) |
| Alcohol consumption |  |  |  |  |  |
| Never | 14.8 | 1.00 |  | 38.8 | 1.00 |
| ≤1 drink per day | 10.5 | 0.57(0.44-0.72) |  | 29.9 | 0.61(0.52-0.72) |
| 2 drinks per day | 16.7 | 1.19(1.07-1.31) |  | 34.6 | 1.33(1.23-1.44) |
| Eat fried foods |  |  |  |  |  |
| Never | 16.3 | 1.00 |  | 40.6 | 1.00 |
| Yes | 10.3 | 0.67(0.61-0.75) |  | 26.5 | 0.73(0.68-0.78) |
| Have a diet low in fat/calories | |  |  |  |  |
| No | 16.3 | 1.00 |  | 38.9 | 1.00 |
| Yes | 12.1 | 0.66(0.61-0.72) |  | 34.7 | 0.77(0.72-0.82) |

^a^ Logistic regression models were used to adjust for all other variables in the table. CI, confidence interval; OR, odds ratio

^b^ Using WHO criteria for Chinese (general obesity: BMI≥27.5 kg/m^2^; abdominal obesity: WC≥90 cm for men, WC≥80 cm for women)

^c^ 100 RMB (Chinese yuan)=6.35 US $
